# A replication analysis of foot-and-mouth disease virus in swine lymphoid tissue might indicate a putative carrier stage in pigs

**DOI:** 10.1186/1297-9716-42-22

**Published:** 2011-02-07

**Authors:** Teresa Rodríguez-Calvo, Fayna Díaz-San Segundo, Marta Sanz-Ramos, Noemí Sevilla

**Affiliations:** 1 Centro de Investigación en Sanidad Animal, (CISA-INIA), Ctra. Algete-El Casar s/n, 28130 Valdeolmos, Madrid, Spain; 2 Plum Island Animal Disease Center, North Atlantic Area, Agricultural Research Service, U.S. Department of Agriculture, Greenport, New York, 11944, USA; 3 Centro de Biología Molecular Severo Ochoa (CSIC-UAM) Cantoblanco, 28049 Madrid, Spain

## Abstract

Foot-and-mouth disease virus (FMVD), one of the most contagious viruses of cloven-hoofed animals, may cause a prolonged, asymptomatic but persistent infection in ruminants, named the "carrier state". However, it remains an open question whether this carrier state occurs in pigs. Here we present quantitative analyses of the duration of FMDV RNA and infectivity in lymphoid and epithelial tissues in experimentally infected pigs with FMDV C-S8c1. The data indicated that although FMDV RNA remained in blood until day 14 post-infection (pi), viremia was cleared by day 7 pi. However, all tissues tested were positive for FMDV until day 14-17 pi. Interestingly, the specific infectivity of FMDV in these tissues was in some cases even higher than the FMDV C-S8c1. We therefore propose that a "pseudopersistent state" may occur in pigs in which virus replicates in lymphoid tissues for a prolonged period of time, thereby representing a potential source of virus.

## Introduction

Foot-and-mouth disease (FMD) is one of the most contagious and economically devastating viral diseases of cloven-hoofed livestock (reviewed in [1-3]). The disease is characterized by the formation of vesicles on the feet, mouth, tongue, and snout, with most of the animals developing viremia, that results in high morbidity but low mortality in adult animals [4]. The infectious agent, FMD virus (FMDV), is a member of the *Aphtovirus *genus of the *Picornaviridae *family, and contains a single-stranded positive-sense RNA genome of about 8 500 nucleotides [5]. FMDV shows high genetic and antigenic variability, which is reflected in the seven serotypes and multiple subtypes reported to date [6]. In the field, this heterogeneity is reflected by the lack of cross-protection even between intraserotype variants [7].

The virus elicits a rapid humoral response in both infected and vaccinated animals, and the infection is eliminated within 7 days post-infection (dpi) [4]. Disease control is achieved by vaccination with chemically inactivated whole-virus vaccine that only provides short-term protection [8]. Many FMD-free countries have included in their national FMD contingency plans the option of using emergency vaccination to avoid the stamping out. However, the response of pigs to emergency vaccination is generally less efficient than that of the other livestock species [9], and therefore, there is a risk that the disease control measures could be compromised. This would be especially important in areas of high pig density where the virus may spread even beyond the areas under restriction. Another important issue in the control of FMD is persistent infections that occur following clinical or sub-clinical disease in both vaccinated and non-vaccinated animals [10]. These persistently infected animals (the so-called carrier state) may be an important source of further outbreaks. In FMDV persistently infected cattle, defined as animals from which live-virus can be recovered 28 dpi [11], the virus can only be found in the nasopharynx associated to the basal layers of the epithelia [12] after clearing from blood and all affected sites. In contrast to other viruses, the mechanisms of establishing persistence in FMDV infection have not been described, although immune responses, both cellular and humoral, and cytokine responses have been suggested as critical components of these mechanisms [1]. A recent study suggested that FMDV persists in the light zones of germinal centres in lymph nodes associated with the pharyngeal region [13], although they did not recovered infective virus from these tissues.

Even though the carrier state has been well characterized in ruminants, some studies have tried to define it in pigs [14,15], with only one report that supports the finding of carrier pigs for FMDV [16]. In this study they did not recovered live-virus but viral RNA in serum after 28 dpi. The use of more sensitive techniques for quantifying FMDV, such as quantitative RT-PCR, has allowed more detailed analyses of FMDV replication in different animals [17,18]. Recently, it has been reported that FMDV RNA can be detected in pig tissues over a period of 28 days following initiation of infection [19]. However, the main question, still unresolved, is whether this viral RNA reflects amount of infectious virus, or is only the result of previous replication of FMDV unable of yielding FMDV infectious particles. Furthermore, it would be interesting to define which tissues are involved in the establishment of persistently infected animals. Here, we describe the recovery of infectious FMDV from several pig tissues over a 17 days period, suggesting that at least during this time, virus might persist in important lymphoid tissues in spite of an ongoing potent humoral response in these animals.

## Materials and methods

### Animals, virus, and experimental design

Twenty Large White × Landrace pigs female 9 weeks old, clinically healthy and free of antibodies against African swine fever virus, classical swine fever virus, Aujeszky disease virus, FMDV, swine vesicular disease virus, and porcine reproductive and respiratory syndrome virus were used for this study. The animals were housed in isolation at the Centro de Investigacion en Sanidad Animal in Valdeolmos (CISA-INIA), Spain. Sixteen animals were inoculated by the intradermal route in the coronary band of the right front limb with 10^5 ^PFU of FMDV C-S8c1 in 0.5 mL of phosphate-buffered saline (PBS). FMDV C-S8c1 is a plaque-purified derivative of natural isolate C1-Sta Pau-Spain 70, a representative of the European subtype C1 FMDV [20]. The animals were slaughtered in batches of two animals at 1, 2, 3, 5, 7, 10, 14, and 17 dpi, except on day 3 where two animals were found dead and only one was sacrificed. Four pigs were used as uninfected controls, housed in different rooms, and sacrificed at the end of the experiment: two were non-inoculated controls and two received an injection of 0.5 mL sterile PBS in the coronary band of the right front limb. All experiments with live animals were performed under the guidelines of the European Community (Directive 86/609/EEC) and were approved by the site institutional animal care and use committee.

### Cell infection

The origin of the BHK-21 cells, procedures for infections of cell monolayers, and plaque assays with FMDV have been described previously [20,21]. BHK-21 cells were infected in six-well plates with the supernatant of the homogenized tissues in PBS. Viral attachment was performed during 1 h at 37°C in 5% CO2. After 1 h adsorption, the cells were overlaid with DMEM containing 5% foetal calf serum, 0.5% agar and DEAE-dextran (0.045 mg/mL). Plaques were visualized 48 h post-infection (pi) by crystal violet staining of fixed cells. The infection of confluent BHK-21 cell monolayers with tissue homogenate supernatants in liquid medium was performed by removing the cell culture medium and adding the supernatant onto the cell monolayer. Virus was adsorbed to cells for 1 h at 37°C in 5% C02 with gentle rocking every 15 min; then the cells were overlaid with DMEM containing 0.5% foetal calf serum. Infections were allowed to proceed until cytopathology was complete, or in case of lack of cytopathology, the supernatant of this infection was used to infect fresh BHK-21 monolayers.

### Tissue samples and histopathological techniques

Animals were anesthetized with Tiletamina and Zolacepam (5 mg/kg) and Atropina (100 mg/kg), and painlessly euthanized with a lethal dose of sodium pentobarbital (Dolethal^®^, Vetoquinol, Madrid, Spain). Tissue samples were collected and fixed in 10% buffered formalin. After fixation, samples were dehydrated through a graded series of alcohol to xylol and embedded in paraffin wax. 4 μm sections were mounted onto electrostatically charged glass slides (SuperFrost Plus, Fisher Scientific, Worcester, MA, USA), re-hydrated, haematoxylin-eosin stained and coversliped using routine methods. Sections were examined in a Leica DFC320 microscope and images were captured with a DFC Twain camera software.

### RNA extraction

Tissue samples were collected, half of each tissue alone and the other half were immediately put into RNAlater (Ambion, Austin, TX, USA), a tissue storage reagent, and stored at -70°C until required. Tissue samples were harvested weighed and homogenized using the "Tissue Lyser" (Qiagen, Valencia, CA, USA) half of each tissue in PBS and the other half in Trizol (Invitrogen, Carlsbad, CA, USA). RNA was extracted by treatment with Trizol (Invitrogen) from swine tissues according to the instructions of the manufacturer. The level of viral RNA was then measured by a quantitative RT-PCR assay.

### Viral RNA quantification

FMDV RNA quantification was performed by real time RT-PCR using the LightCycler instrument (Roche, Basel, Switzerland) and the RNA Master SYBR green I kit (Roche) as specified by the manufacturer. Quantification was relative to a standard curve obtained with known amounts of FMDV C-S8c1 RNA, using a procedure that has been described previously [22,23]. The primers used to quantify total FMDV RNA in tissues were described in [24].

## Results

### Evolution of FMDV lesions in the coronary band epithelium

**Figure 1:**
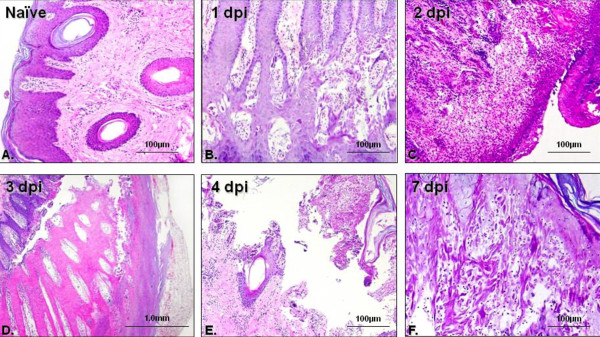
**Lesion evolution in infected animals**. Skin from the coronary band (different location that the inoculation site) of infected pigs was collected at different times-points, fixed, paraffin-embedded and routinely stain with haematoxylin and eosin. The figures represent the lesions observed in all the animals at each time-point.

Twenty pigs were infected with 10^5 ^PFU of FMDV C-S8c1 in the coronary band and four pigs were used as uninfected controls. The infection resulted in severe, acute symptoms including fever and vesicle formation in the mouth, snout and coronary bands on the feet, not only at the inoculation site but also on other feet. Two animals were slaughtered at days 1, 2, 5, 7, 10, 14 and 17 pi. at day 3, two animals were found dead and only one animal was sacrificed. Blood and several tissue samples were collected: spleen, thymus, inguinal lymph node (ING), pre-scapular lymph node (PRE), mesenteric lymph node (MES), mediastinic lymph node (MED), retro-pharyngeal lymph node (RTF), tonsil and skin (at the coronary band), and divided in two pieces, one processed for histopathological analysis and the other frozen to perform RNA extraction and virus isolation. Lesions were initially examined by light microscopy using 4 μm thick sections stained with haematoxylin and eosin. Histopathological changes in the skin were observed as early as 1 dpi, although macroscopical lesions were not observed yet. At this time point, the stratified squamous epithelium showed ballooning degeneration, increased cytoplasmic eosinophilic staining of the cells in the stratum spinosum and acantholysis (Figure 1B) as compared with healthy skin sections (Figure 1A). At 2 dpi we observed oedema within the dermis and an intense granulocyte and mononuclear cell infiltration (Figure 1C). This early stage was followed by necrosis and subsequent vesicle formation by separation of the epithelium from underlying tissue filling the cavity with vesicular fluid at 3 dpi (Figure 1D). Later on, the lesions developed further into larger vesicles with high production of vesicular fluid that could be broken by physical trauma (Figure 1E). By 7 dpi, the epithelium was starting to reconstitute its architecture (Figure 1F), although this process took up to 14 days where crust could still be observed (data not shown).

### Virus replication in the epithelium of the coronary band

**Figure 2:**
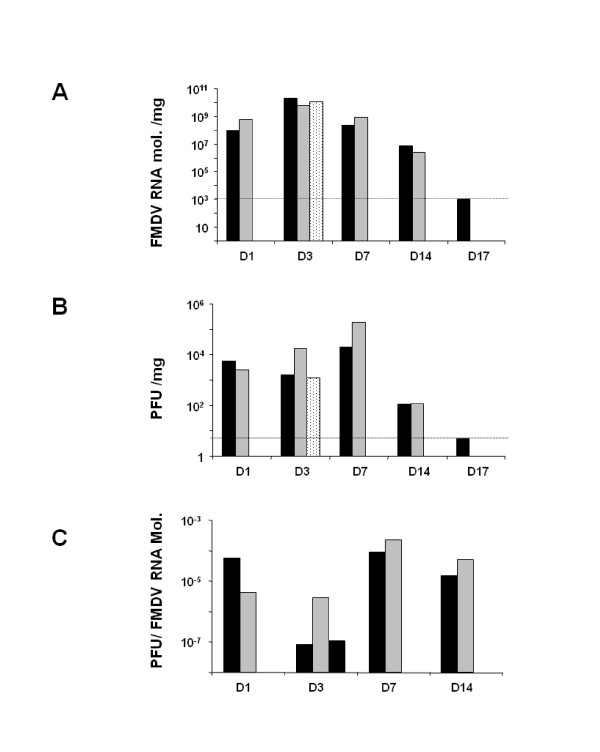
**FMDV load in epithelium**. A. Viral particles were detected by quantitative RT-PCR and expressed as FMDV RNA molecules/mg of tissue. The dotted line corresponds to the detection limit of the technique. B. Viral load is expressed as PFU/mg of tissue after titration on BHK-21 cells. Each bar corresponds to one animal. At day 3 pi two animals were found dead and one animals was euthanized, reason why the graphic has 3 animals for this time-point. C. Specific infectivity of each pig is indicated as PFU/FMDV RNA molecules, determined as described in Materials and methods. Each bar corresponds to one pig. Day 17 pi is not included because it was negative for FMDV detection. * No viral RNA or infectious virus was detected at 17 dpi

The manifestation of lesions in skin (vesicles) has long been recognized as a key feature of disease in pigs and cattle caused by FMDV. The coronary band epithelium is one of the main sites of FMDV replication in vivo, as well as the inoculation site for experimentally infected pigs. To determine viral load in epithelium, tissue homogenates were analysed by quantitative RT-PCR and plaque assay on BHK-21 cells. Sampling of the primary lesion that develops at the site of inoculation revealed substantial concentrations of viral genome by 1 dpi (average of 3.5 × 10^8 ^viral RNA molecules/tissue mg), which increased gradually to 10^10 ^RNA molecules by 3 dpi (Figure 2A). At later time points, the concentration of FMDV RNA decreased until 17 dpi, when viral RNA could no longer be detected. Infectious virus was also readily isolated from the skin at the inoculation site on all days post-inoculation but 17 dpi. Interestingly, although the highest FMDV RNA concentration was obtained at 3 dpi, the highest amount of infectious virus, measured by plaque assay, was observed at 7 dpi (Figure 2B). Based on these data, we can determine the specific infectivity of viral RNA found at different times post-infection in skin as the ratio between number of viral genomes and PFU in a fixed amount of tissue. The specific infectivity of FMDV RNA isolated at epithelium in the coronary band ranged from 3 × 10^-5 ^PFU/FMDV RNA mol. at 1 dpi, declining at 3 dpi (1 × 10^-8 ^PFU/FMDV RNA mol.), a second increased at 7 dpi (1.5 × 10^-4 ^PFU/FMDV RNA mol.), and finally declining again at 14 dpi (3.2 × 10^-5 ^PFU/FMDV RNA mol.) (Figure 2C). Thus, the infectivity in epithelium remains high even at time points when skin lesions are resolving or completely healed.

### Detection of FMDV RNA and infectivity in serum

**Figure 3:**
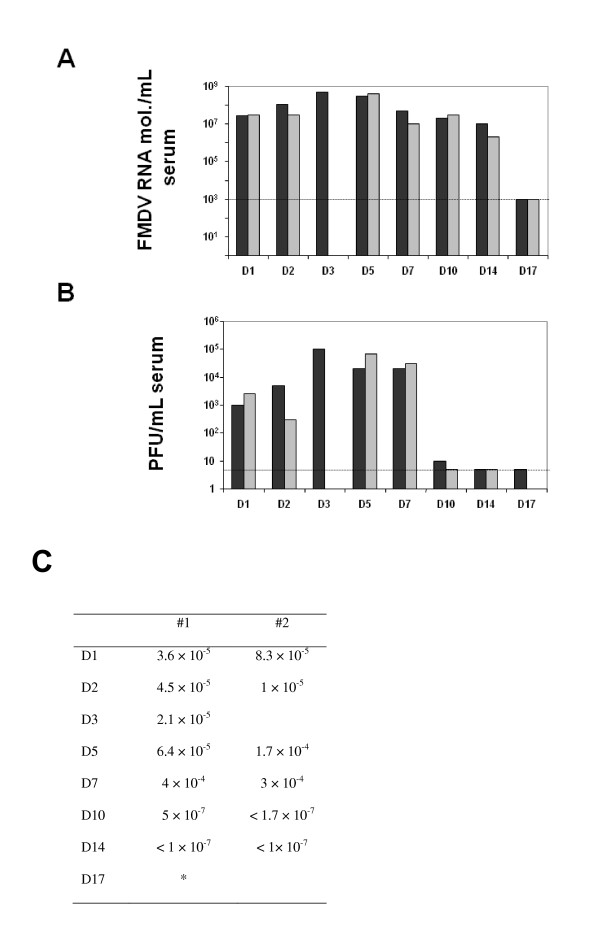
**FMVD replication in serum**. Two pigs were bled at each time point. FMDV has been quantified by quantitative RT-PCR and titration in BHK-21 cells. A. It is represented the number of FMDV RNA molecules/mL of serum. The dotted lined indicates the detection limit of the technique (10^3 ^FMDV RNA molecules). B. Bar graph indicated the number of PFU/mL of serum quantitated by plaque assay on BHK-21 cells (see Materials and methods). Each bar represents one animal. At 3 dpi one animal of the group was found dead, reason why we did not collect serum from that animal. The dotted line indicates the detection limit of the technique (5 PFU). Each bar represents one animal. C. The specific infectivity is indicated per each animal. It is expressed as the number of PFU per viral RNA molecule.

The viremia of FMDV-infected swine was assessed, initially, using quantitative RT-PCR to detect viral genomes and, subsequently, by virus isolation and plaque assay in BHK-21 cells to confirm the presence of infectious virus. Using RNA extracted from serum, FMDV RNA was first detected at 1 dpi (average of 2.5 × 10^7 ^FMDV RNA molecules/mL) (Figure 3A). The viral load in serum peaked at 3 dpi (5 × 10^8 ^FMDV RNA molecules/mL). Viral RNA was observed until 14 dpi (average of 6.3 × 10^6 ^FMDV RNA molecules/mL), while it was no longer detected by 17 dpi. In order to confirm the infectivity of these viral RNAs, plaque assays on BHK-21 cells were performed. The data show infectious virus at 1 dpi (average 1.75 × 10^3 ^PFU/mL), with the highest viral load at 3 dpi (1 × 10^5 ^PFU/mL) (Figure 3B). While the conventional FMDV plaque assay showed viremia only in one animal at day 10 dpi (10 PFU/mL, with a detection level of our assay of 5 PFU/mL) and did not detect it at 17 dpi, the quantitative RT-PCR was able to detect viremia until 14 dpi. Thus, viral RNA can be detected in serum although no infectious virus was detected. The specific infectivity of FMDV RNA calculated in serum ranged from 1.2 × 10^-4 ^PFU/genomic RNA molecule at 5 dpi to 3.3 × 10^-7 ^at 10 dpi PFU/genomic RNA molecule (Figure 3C). Therefore, in spite of the high amounts of viral RNA detected in serum for at least 14 dpi, the specific infectivity of this FMDV RNA decreases by day 10 pi to reach values that are undetectable or at least 360-fold lower than at the peak of viremia.

### Viral infection clearance from lymphoid tissue

**Figure 4:**
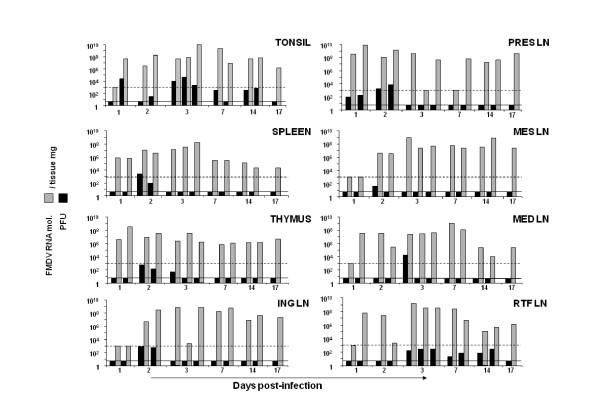
**FMDV load in lymphoid tissues**. Each graph indicates the FMDV RNA molecules (grey bars) or PFU (black bars) per mg of tissue as indicated: tonsil, spleen thymus inguinal lymph node (ING LN), pre-scapular, lymph node (PRES LN), mesenteric lymph node (MES LN), mediastinal lymph node (MED LN) and retro-pharyngeal lymph node (RTF LN). The dotted line indicates the detection limit for FMDV RNA (10^3 ^FMDV RNA molecules) and the black line indicates the detection limit of the FMDV titration (5 PFU/mg of tissue). Each bar corresponds to one animal.

Following primary infection, FMDV is disseminated throughout the body. Virus spreading to lymphoid organs, which has not been well characterised in FMDV pathogenesis in swine, could represent an important route for the establishment of a viral reservoir. To address this question, we have determined viral replication by plaque assay on BHK-21 cells and quantitative RT-PCR during 17 days pi in tonsil, spleen, thymus and several lymph nodes (LN) (ING, PRES, MES, MED and RTF). Viral RNA was detected in all tissues at different times post-inoculation, even at day 17 pi, when the viremia was completely cleared (Figure 4). Most of the lymph nodes showed high amounts of viral RNA at later times pi (days 14 and 17) except for the MED and RTF LN where the amount of viral RNA detected was lower. Interestingly, tonsil was the only tissue in which the presence of infectious virus was observed at all studied time-points until 14 dpi (Figure 4). Meanwhile, other tissues did not show infectious virus by plaque assay but high amount of viral RNA could be detected. This might indicate that the amount of FMDV particles was very low (below our detection limit, 5 PFU/tissue mg). To address this possibility, the tissue samples homogenates were used to infect BHK-21 cells in liquid medium during 3 days, and cytopathic effect (cpe) was observed during this time. All tissues that were positive for FMDV RNA by RT-PCR at different times post-infection and that were negative by plaque assay to detect virus replication showed cpe in BHK-21 cells (Table 1). This suggests that although the level of virus was below the detection limit for the plaque assay, infectious virus was recovered in all tested tissues, even at 17 dpi (the latter time in the study). As a value of virulence, specific infectivity was calculated in those tissues in which viral load had been quantitated by plaque assay (Table 1). Among all the studied tissues, tonsil deserves special attention since specific infectivity was still high (average of 8.5 × 10^-6 ^PFU/RNA mol.) at 14 dpi. Taken together these data suggest that not only viral particles were present in tissues even at times when viremia had been completely cleared but also that these virions were highly infectious.

**Table 1:** Specific infectivity of FMDV RNA of different lymphoid tissues

	Tonsil	Spleen	Thymus	ING	RTF	MED	MES	PRE
**1**	ND^a ^(+++)^b^	ND (+)	ND (-)	ND (-)	ND (-)	ND (-)	ND (-)	5 × 10^-7^
	6 × 10^-4 c^	ND (+)	ND (+)	ND (-)	ND (+)	ND (-)	ND (-)	7 × 10^-8^

**2**	ND (+)	2 × 10^-4^	7 × 10^-5^	1 × 10^-4^	ND (+)	ND (+)	1 × 10^-5^	5 × 10^-6^
	2 × 10^-7^	2 × 10^-5^	4 × 10^-6^	1 × 10^-6^	ND (+)	ND (+++)	ND (+++)	2 × 10^-6^

**3**	2 × 10^-4^	ND (+++)	2 × 10^-5^	ND (++)	1 × 10^-7^	4 × 10^-4^	ND (+++)	ND (+)
	5 × 10^-4^	ND (+++)	ND (++)	ND (+)	ND (++)	ND (+++)	ND (+++)	ND (-)
	3 × 10^-7^	ND (+++)	ND (++)	ND (++)	ND (+)	ND (+++)	ND (+++)	ND (+)

**7**	2 × 10^-7^	ND (+++)	ND (+++)	ND (+++)	ND (++)	ND (+++)	ND (+++)	ND (+)
	ND (++)	ND (+++)	ND (+++)	ND (+++)	ND (++)	ND (+++)	ND (+++)	ND (+++)

**14**	7 × 10^-6^	ND (+++)	ND (+++)	ND (+++)	ND (+++)	ND (+++)	ND (+++)	ND (+++)
	1 × 10^-5^	ND (+++)	ND (++)	ND (++)	ND (+++)	ND (+++)	ND (+++)	ND (+++)

**17**	ND (+++)	ND (+++)	ND (+++)	ND (+++)	ND (+)	ND (+++)	ND (+++)	ND (+)

It is indicated the specific infectivity of FMDV RNA at different times post-inoculation (right columna) from tonsil, spleen, thymus and several lymph nodes [inguinal (ING), retropharyngeal (RTF), mediastinal (MED), mesenteric (MES), and prescapular (PRES)].

^a ^ND: not determined.

^b ^Cythopatic effect: BHK-21 cells were infected with homogenate from tissues and cytophatic effect (cpe) was evaluated at 48 h post-infection. (-), no cpe; (+), 10-20% of cpe; (++) 50% of cpe; (+++) 100% cpe.

^c ^Specific infectivity is expressed as the number of PFU per viral RNA molecule.

## Discussion

Two critical determinants of FMD pathogenesis are the ability of the virus infection to spread through the different tissues and, the clearance rate by the host immune response. This is of particular relevance regarding the possibility of FMDV persistence in pigs. In the present study we show evidences that, at least during 17 dpi, FMDV can be isolated from different lymphoid tissues, not only as viral RNA but also as infectious virus. FMDV was also detected in coronary band epithelium even at time points when viremia was already cleared. To the best of our knowledge, this is one of the first reports where infectious virus has been isolated from swine lymphoid tissues, at later times than 3-5 dpi. The results of these studies have important implications for understanding both the viral spread in pigs and the likelihood for the virus to persist in the animals.

The evolution of the epithelial lesions suggests that one of the first sites of viral replication is the stratum spinosum, spreading later on to all epithelial layers. The appearance of lesions is correlated with the presence of FMDV RNA in coronary band epithelium in which viral load was within the range of 10^8^-10^10 ^molecules/mg. However, by day 17 pi no FMDV RNA was detected, indicating that virus was cleared from epithelium at this time. Interestingly, the specific infectivity of FMDV RNA in this tissue remains high at 14 dpi (3.2 × 10^-5 ^PFU/FMDV RNA mol as compared with the specific infectivity showed by the virus used for challenge, FMDV C-S8c1, 2.7 × 10^-6 ^PFU/FMDV RNA [24]), suggesting that although the amount of virus detected is not remarkable, the infectivity of this virus is even higher than that of the parental virus. Alexandersen et al. [14] proposed that a cycle of FMDV replication in pigs is of 12-24 h duration, initially replicating in epithelial cells and resulting in a significant amplification of the virus, producing high viremia and the appearance of clinical disease. After several replication cycles, in which the virus infects other susceptible cells, the host immune response is likely to prevent the development of higher level of virus. The fact that viremia has been cleared but virus was still found in epithelium might indicate that the virus is sequestrated from the circulation trying to escape immune surveillance as has been previously suggested for cattle [13]. We are currently investigating whether this might be the case in pigs. On the other hand, a number of experiments have been carried out in cattle, in which FMDV transmission from carriers to susceptible animals was studied [25,26]. Although virus transmission from carriers to susceptible animals in experimental model has not been described it would also be interesting to rule out the possibility of transmission of disease from convalescent pigs to other natural susceptible animals.

FMDV replication in tonsils has been found until the last day tested in this work (17 dpi), being unexpectedly high at day 17. Zhang et al. [19] described the detection of FMDV RNA in pig tonsil at 28 dpi, although no infectious virus was isolated in this report. In addition, Carrillo et al. [27] described virus isolation from pig tonsil at day 26 post-contact. These results, together with our data, indicate that virus is likely to persist in pigs to some degree following infection, resembling what has been described for cattle [1,28] but not yet described for pigs. Nevertheless, it would be interesting to further investigate the presence of infectious virus in tonsil at later time points as a mechanism of persistence in pigs.

The predilection for the palatine tonsil showed in our work correlates with high viral replication in RTF LN, in which infectious virus can be detected at 14 dpi, and viral RNA until 17 dpi. Given that the RTF LN constitutes the draining pathway of the palatine tonsil [29], the high amount of virus in both anatomical locations until late during the infection might indicate that virus is being shed from the tonsil to the RTF LN. Since all the oropharynx area has been related to persistence of FMDV in cattle [1], our data support the possibility of a similar persistent stage in pigs, although additional data regarding later times post-inoculation are needed to confirm this hypothesis.

Some reports [16] have pointed out the presence of FMDV RNA in blood for a period of at least 28 dpi. In our study, the spleen and thymus, two major lymph organs, showed high amounts of FMDV RNA at 17 dpi and replicating virus measured by infection in BHK-21 cells. It is unlikely that the virus is produced elsewhere and filtered in these organs since by this time point in our study the viremia is completely cleared. A similar pattern is found in lymph nodes, in which the amount of FMDV RNA is high by 17 dpi and infectious virus can be rescued in all lymphoid tissues. The fact that FMDV RNA was rescued in these tissues suggests that viral replication has taken place at some point during viral spreading. One possibility could be the sequestration of immune complexes of FMDV particles within lymphoid tissue that has been suggested as a possible source of infectious material detected in tissues samples [13,30,31]. Different cell types such as B cells, macrophages and dendritic cells (DC) are able to support virus replication cycle at some level in the presence of high amounts of neutralising antibodies [31-33], and these cell types may also act as a source for viral spreading to other sites. This mechanism could also account for the detection of viral RNA 28 dpi in blood. Indeed, these cell types might act as a reservoir of virus in a replicative or non-replicative stage that eventually gets activated giving as a consequence a new virus production and, possible, an outbreak. Recently, FMDV RNA has been detected in germinal centers (GC) of cattle lymphoid tissues, suggesting that GC might be a reservoir area for infectious virus in lymphoid tissue [13]. More interestingly, DC network might trap virus and serve as a repository for maintenance of persistence. Nevertheless, to distinguish sequestration of non-replicating virus from replicating virus (that eventually might be a source of infectious virus), minus strand RNA have to be measured. We are currently carrying out experiments to determine the role of DC in this pseudopersistence stage described for pigs.

Evolutionary patterns of virus replication and distribution in lymphoid tissue during 17 days pi with FMDV in pigs has shown that replication, or at least FMDV RNA detection, remains elevated in these lymphoid tissues despite the fact that viremia is significantly low or cleared. However, identification of infected internal structure from each tissue and of infected cell types deserves further investigation. These findings have implications for a putative persistence in pigs although with clear differences to that observed in cattle. Indeed, a longer follow-up time of infectivity in pigs will be needed to determine a true carrier state as described in cattle. Nevertheless, based on the evidences presented here, the paradigm that FMDV does not persist in pigs should be reconsidered.

## Competing interests

The authors declare that they have no competing interests.

## Authors' contributions

TR, carried out most of the experiments described in the manuscript and participated in the design of the study; FD, participated in the design of the study and carried out some of the experiments; MS, participated in the initial design and experiments of the real time PCR described in the manuscript; NS, conceived of the study, and participated in its design and coordination. All authors read and approved the final manuscript.
